# Microbiological Safety and Shelf-Life of Low-Salt Meat Products—A Review

**DOI:** 10.3390/foods11152331

**Published:** 2022-08-04

**Authors:** Coral Barcenilla, Avelino Álvarez-Ordóñez, Mercedes López, Ole Alvseike, Miguel Prieto

**Affiliations:** 1 Department of Food Hygiene and Technology, Faculty of Veterinary Medicine, University of León, 24071 León, Spain; 2 Institute of Food Science and Technology, University of León, 24007 León, Spain; 3 Animalia—Norwegian Meat and Poultry Research Centre, NO-0513 Oslo, Norway

**Keywords:** microbiological safety, low-salt meat products, shelf-life, water activity

## Abstract

Salt is widely employed in different foods, especially in meat products, due to its very diverse and extended functionality. However, the high intake of sodium chloride in human diet has been under consideration for the last years, because it is related to serious health problems. The meat-processing industry and research institutions are evaluating different strategies to overcome the elevated salt concentrations in products without a quality reduction. Several properties could be directly or indirectly affected by a sodium chloride decrease. Among them, microbial stability could be shifted towards pathogen growth, posing a serious public health threat. Nonetheless, the majority of the literature available focuses attention on the sensorial and technological challenges that salt reduction implies. Thereafter, the need to discuss the consequences for shelf-life and microbial safety should be considered. Hence, this review aims to merge all the available knowledge regarding salt reduction in meat products, providing an assessment on how to obtain low salt products that are sensorily accepted by the consumer, technologically feasible from the perspective of the industry, and, in particular, safe with respect to microbial stability.

## 1. Introduction

Consumers are increasingly demanding foods with a low salt content, which are perceived as healthier and fresher. Reducing salt intake may lower many commonly associated risks, including high blood pressure, cardiovascular disease, stroke, and coronary heart attack, and has been identified by the World Health Organization (WHO) as one of the most cost-effective measures that countries can take to improve health outcomes [1].

The use of salt in food manufacturing has been traditionally related to its ability to keep foods edible for longer periods of time, which would allow manufacturers to extend their consumption in periods of scarcity [2]. In ancient times, salt was used as a preservative in many foods such as fish, meat, and dairy products, due to its preservative quality and sensorial properties [3]. Indeed, as a highly estimated trading commodity, it is argued that the word salary derives from the Latin salarium, i.e., money given to Roman legionnaires to buy salt [4].

To ensure safe foods with an extended shelf-life, classical food preservation processes (thermal processing, drying, salting, freezing, etc.) usually impose extreme physical and/or chemical barriers to prevent the growth of, or to inactivate, spoilage and pathogenic bacteria [5]. Salting has been traditionally used in many instances as a simple and inexpensive method of preservation since it does not need sophisticated equipment and imparts suitable sensory properties to the product. It was not until the 1950s that cold storage was introduced in private households, reducing the need for salt as a basic additive. Still, this technological advance did not cause a reduction in the amount of salt in the diet. Indeed, the consumption of salt increased simultaneously with the expansion of industrially processed foods, illustrating consumers’ strong appreciation and preference for a salty taste [6,7,8]. These dietary changes resulted in less individual-level control of sodium intake and, for many, a chronic excess of sodium intake from non-discretionary sources [4]. In addition, salt is very appreciated from a technological point of view due to its various properties. Considered as a multifunctional ingredient, salt is used to enhance flavor, mask off-flavors, improve food structure and texture, promote water holding capacity, and reduce water activity, which finally leads to a preservation action [9].

The issue of salt and how it relates to health and food safety has been frequently dealt with by regulatory agencies [10,11,12,13,14,15]. The food industry faces big challenges derived from salt reduction, and, although it has met the challenge, there is still a need to balance salt reduction in products with consumers’ taste preferences, the microbiological safety of the products, and food technological constraints [16,17]. Some producers have already reduced the salt content successfully [18,19,20], but further reductions depend on salt replacers or additives that work as effective substitutes [16,21,22]. However, salt concentrations commonly used in fermented meats inhibit the growth of undesired microorganisms, and, at the same time, promote the growth of more salt-tolerant lactic acid bacteria [23,24,25], while high salt concentrations may also inhibit the growth of starter cultures in fermented meats, as happened in a sausage made with 5% instead of 2.5% NaCl [26] or with 2.4% instead of 1.01% [27]. Paradoxically, food scares regarding E-numbers and the “clean label” trends are significant obstacles for the development of healthier low-sodium products [14,27]. Thus, research is still on-going regarding low-sodium meat products, which indicates that the direct reduction of this component to reach authorities’ recommendations in this type of products is not an easy target [28].

The reduction of salt in food products and the increasing use of replacers (e.g., KCl) as an alternative to NaCl may represent potential safety risks arising from such reformulations. While the consequences of sensory and technological properties have received considerably more attention [9,29,30,31,32,33,34], salt reduction and the use of replacers as an alternative to NaCl may represent potential safety risks [17]. Before reducing the salt content of meat products or replacing it with alternative ingredients, it is necessary to assess the microbiological stability of original and reformulated meat products by studying the consequences for safety and quality [8]. Therefore, the aim of this article is to review the mechanisms of microbial inhibition by salt, to evaluate the microbiological risks deriving from a low level of NaCl in meat products and to critically review the available management measures aimed at minimizing the risks associated with this type of reformulated product.

## 2. Health Risks Associated with Salt Consumption

The ingestion of elevated levels of sodium chloride is associated with increased blood pressure and hypertension, induced cardiovascular disease (CVD) and stroke [28,29]. The increased risk of cardiovascular events associated with a higher sodium intake (>5 g/d) is most prominent in those with hypertension [30]. However, increasing evidence has shown that a high intake of salt is also a risk factor for otherwise healthy people [31,32]. Current evidence from prospective cohort studies suggests that the lowest risk of cardiovascular events and death occurs in populations consuming an average sodium intake ranging from 3 to 5 g/d. Sodium reduction seems to increase heart rate independently of the reducing effect on the baseline blood pressure of sodium. Hence, lowering the sodium intake is best targeted at populations with hypertension who consume high-sodium diets [33,34,35]. The long-term effect of salt intake in doses higher than the physiological need is mainly increased blood pressure with age. There is also strong evidence that risk is reduced when salt intake is lowered, independent of age. Therefore, there is a health motivation for almost everybody to control and reduce their salt intake [29].

Physiologically, sodium levels are strictly controlled in the bodies of humans and animals. Sodium is the most important and prevalent metal ion in the body’s tissues. Indeed, it is essential for homeostasis, blood pressure maintenance, water holding, and neural transmission, among others [36]. The salty taste reflects the Na^+^ ion concentration in the mouth, which in turn causes a positive response in the brain, and hypertonic blood concentrations seem to be preferred [37]. The daily salt intake across the world has typically varied between 9 and 12 g NaCl [38]. However, the World Health Organization recommends a daily consumption of 5 g NaCl, equivalent to approx. 2 g/day of sodium [13].

Estimates of the global burden of disease from high systolic blood pressure are receiving increased attention [39]. In the USA, it was estimated that a 3 g/day salt reduction would save 194,000–392,000 quality-adjusted life-years and $10–24 billion in healthcare costs annually [40]. In Europe, the Framework for National Salt Initiatives was developed in 2008 with the overall goal of contributing towards a reduced salt intake at population level. Thus, the initiative identified five key elements to focus action on, which included (i) data availability, (ii) benchmarks and major food categories, (iii) raising public awareness, (iv) developing reformulation actions with industry/catering, and (v) monitoring and evaluation of actions. Overall, the EU framework set a realistic benchmark of a minimum 16% reduction over a 4-year period in all food categories [41]. Remarkably, countries with a higher sodium intake, i.e., the Czech Republic, Slovenia, and Hungary, exhibit a higher prevalence of hypertension [42]. A decrease of salt intake to 5 g per day is expected to substantially reduce the burden of cardiovascular disease and mortality. In Finland, the salt consumption has been considerably reduced, while the salt intake in other countries such as Poland has remained relatively high. Indeed, a reduction in salt intake to reach the WHO population nutrient goal would reduce the prevalence of stroke around 10.1% in Finland and 23.1% in Poland [43]. Regarding cardiovascular diseases, a 17% decrease is expected if the WHO target is reached, which will prevent an estimated 4 million deaths annually worldwide [42]. The UK and Ireland are other successful examples that have been following salt-reduction strategies among different food products [8]. Japan, which is a country well-known for its low mortality rates, reduced its salt intake from 14.5 g in 1973 to 9.5 g in 2017, which has led to the reduction in stomach cancer and other cerebrovascular diseases [44]. However, this reduction is still far from the 5 g NaCl recommendation [13]. Nevertheless, the socioeconomic status of individuals plays an important role in the type of diet followed and thereby the amount of sodium ingested [42].

## 3. Functions and Content of Salt in Meat Products

Salt (NaCl) has three main functions in meat products: a preservative effect, the contribution to organoleptic quality attributes (flavor and texture), and a technological function as to provide binding between meat and fat [8,38,45,46].

**The preservation** (extended shelf-life and microbiological stability) of meat products can be attained by lowering the water activity by the addition of a solute and through dehydration by removal of water by simple evaporation. Water molecules are retained among Na^+^ and Cl^−^ ions and thus become unavailable for other functions, such as chemical or enzymatic reactions or to be used by microorganisms [47]. This provokes the inhibition of the spoilage and pathogenic microbiota, which in turn increases the shelf-life and safety of foods [8]. Stringer et al. (2005), evaluated this by modelling the growth capacity of *Aeromonas hydrophila*, *Clostridium botulinum*, *Listeria monocytogenes*, *Yersinia enterocolitica*, and *Bacillus cereus* in two NaCl concentrations in chicken rolls (3.04 and 1.61%), ham (5.57 and 2.81%), and bacon (5.5 and 2.85%). All bacteria presented a greater growth rate under a reduced salt content in all products in the model. Moreover, *C. botulinum* was able to grow in products with 2.85% salt, whereas it was not able to in 5.5% salt [48].

Salt is widely used as a **flavor and palatability enhancer**, since it improves the positive sensory attributes of most foods and helps attenuate bitterness and sweetness, thus being a major determinant for consumer acceptance. When different salt concentrations ranging from 0.8 to 2.2% *w*/*w* were added to pork breakfast sausages, consumers found the most acceptable samples had 1.4% salt [49]. Salting also helps the volatilization of certain molecules in foods, thus intensifying the aroma of the food [45]. Salted meat products such as dry-cured ham are quite popular, because they have unique sensory characteristics [47].

In meat products, salt operates as a **binding agent** between meat and fat. It increases the water-binding capacity, so that the final product shows improved yield, texture, tenderness, and palatability. The salt in meat products at 1.5–2.5% promotes the solubilization and extraction of myofibrillar proteins (actin and myosin) that are insoluble in water alone, therefore being fundamental in the gelation and binding of many restructured meat products [46]. Salting, in combination with processing steps such as blending and tumbling, helps extract these salt-soluble myofibrillar proteins to the meat surface, which is essential for holding pieces of meat together in batters and restructured meats and contributes to forming a gel between meat particles and between meat and fat particles [50]. The solubility degree of myofibrillar proteins is directly depending on the amount of NaCl in the meat product [51].

The range of the salt content in meat products is very large (Table 1), and even similar or equivalent meat products can be elaborated with different concentrations depending on the particular formulations. This suggests that it is feasible to generally reduce the salt content of foods. Meat products are considered the second biggest source, after bakery products, of salt intake in Europe [42]. This is quite outstanding, especially considering that the amount of salt naturally present in fresh meat is very low compared to meat products after processing [8,42]. In an analysis performed on a series of meat products, the results showed an average of 2.14 g NaCl/100 g of product, with the lowest value of 0.84 for turkey breast and the highest of 7.81 for ham [52]. In relation to the intake, in industrialized countries, about 75–80% of salt is ingested in processed foods, and especially meat products, which constitute one of the major sources of sodium in the form of salt or other additives [42,52]. Four food groups include almost 60% of the total ingested salt, i.e., cured meat products (26.2%), bread products (19.1%), cheese (6.7%), and processed ready-to-eat (RTE) foods (4.9%). It is estimated that, in countries such as Ireland or the United States, processed meat products contribute to more than 20% of the daily sodium intake [15,53]. It should be noted that, in cured meat products, sodium can stem not only from common salt, but also from sodium nitrite and the additives sodium ascorbate and sodium erythorbate, which are used as reducing agents. Other possible sources are sodium tripolyphosphate, monosodium glutamate, and hydrolyzed vegetable protein.

### 3.1. Salt as a Chemical Preservative

Meat products are made primarily of muscle meat added with salt and nitrites, which are responsible for the curing. The curing process changes the flavor and color of the meat and improves the shelf-life and safety of the product by inhibiting spoiling microbiota and pathogens, while certain microbial groups such as lactic acid bacteria (LAB) and *Micrococcaceae* are promoted [17,59,60]. The addition of salt and other solutes (salt replacers, sugars, humectants, proteins, etc.), together with the dehydration caused by the removal of water by simple evaporation, decreases water activity (a_w_), reducing or inhibiting microbial and enzymatic activity and, therefore, accomplishing a significant preservation effect. Meat products manufactured with NaCl levels below those typically formulated have usually a shorter shelf-life [8]. For instance, the shelf-life of a reduced-salt bacon (2.3% *w*/*w* NaCl) was 28 days, whereas for the control bacon (3.5% *w*/*w* NaCl) it was 56 days [48]. Certainly, salt is not the only barrier to spoilage microbiota and pathogens present in meat products. The combined use of other preservation hurdles, such as low temperature, acidification [60], antimicrobial compounds [38,61,62,63], limited oxygen availability or HPP treatment [64], contributes to the inhibition of certain microbial groups and causes a shift in the prevailing microbial populations. For example, Michelakou et al. (2021) stated that abusive storage temperatures somehow limited the effect of salt, thus indicating that low temperatures help to hold certain microbial growth [65]. Moreover, potassium lactate allowed a 30% reduction from 4 to 2.8% salt in salami without repercussions in the antimicrobial capacity [62]. Several antimicrobial compounds are under research for future application as antimicrobial food cultures in different products, among them meat products, and quite promising results are being achieved using lactic acid bacteria and their bacteriocins [66,67,68].

### 3.2. Low Water Activity as an Environmental Stress Factor—Molecular Basis of NaCl Action as a Preservative and the Bacterial Adaptive Response against NaCl

Water activity is an indicator of the amount of water that is available for microbial growth and other chemical reactions in a particular food and can be defined as the ratio between the partial vapor pressure of a given food in relation to the vapor pressure of pure water at the same temperature. Considering Raoult’s law, where the vapor pressure is related to the molar fraction of a solute in a solution, the higher the concentration of salt, the lower the a_w_. Generally, an a_w_ of 0.85 is considered the lowest value for any human pathogen bacteria to grow, in accordance with the requirement for *Staphylococcus aureus* toxin production (Table 2 shows the a_w_ required by different bacterial pathogens). However, yeast and mold are able to grow at lower a_w_ levels, and even some rare xerophilous bacteria, which undergo a phenomenon known as anhydrobiosis, can persist in extreme dehydration. Nonetheless, for most foods, the a_w_ is between 0.95 and 0.99 [69]. The a_w_ of a food can be reduced by increasing the concentration of solutes in its aqueous phase through drying, water extraction, freeze drying, etc., or by adding new solutes. Salt is one of those solutes able to reduce a_w_ due to the association of sodium [Na^+^] and chloride ions [Cl^−^] ions with water molecules. Water dissolves salts due to its marked polarity and capacity to form weak hydrogen bonds; the electronegative pole of the H_2_O molecule (oxygen) is attracted to the positively charged [Na^+^], and the electropositive pole (hydrogen) is attracted to the negatively charged [Cl^−^] [70].

Salt produces an osmotic imbalance in microbial cells by extracting water from the cells through the membranes of both food tissues and microorganisms through the process of osmosis. The retraction and reduction of the cytoplasmic volume is a phenomenon known as plasmolysis. Osmotic stress due to a high concentration of solutes can occur in food during manufacturing, forcing microbial cells to cope internally with this adverse environment in order to survive. This event can occur suddenly, after rapid exposure to highly concentrated solutions (e.g., osmotic shock during salting), or progressively, as the product slowly dehydrates (e.g., during ripening). Osmotic shock provokes water efflux and dehydration in the cells, the release of low molecular-weight compounds and cell proteins, and the perturbation of many cellular physiological functions [72,73]. However, the progressive exposure to osmotic stress can allow cells to adapt gradually and maintain homeostasis by means of an adaptive response.

Cellular adaptive response. To protect the cell against the damage inflicted on functions and key molecules such as enzymes and macromolecular structures, bacteria use common adaptive stress pathways in response to a diverse range of adverse environmental conditions (such as dehydration) [74,75,76]. For this purpose, they have evolved adaptive networks, such as biofilm formation, shifts in metabolism, or changes in the cell membranes, that allow them to cope with the challenges of a changing environment [69,77]. There are three basic microbial strategies used to overcome exposure to a low a_w_ environment [78]:

- some cells counterbalance the levels of inorganic ions (usually KCl) to achieve osmotic stability;
- some are able to modify the membrane permeability, structure, and/or composition to protect the cells; and
- some produce or accumulate low-molecular-weight compounds that have the osmotic capacity to counteract the extreme external osmotic pressure. These osmolytes are defined as compatible solutes and are polar, normally uncharged, molecules. Compatible solutes act in the cytosol to counterbalance high external osmolarity, thus preventing water loss from the cell and plasmolysis, without adversely affecting the macromolecular structure and enzymatic functions. These compatible solutes belong to several classes of compounds with some common structural motifs, some amino acid derivatives being particularly important [79].

Bacterial cells usually activate several synergistic response pathways to survive low water-activity environments or hyperosmolarity conditions [69]. It is generally acknowledged that bacteria react to environments of elevated osmolarity by means of a biphasic response, which involves the stimulation of potassium uptake (and its counter-ion glutamate) followed by a dramatic increase in the cytoplasmic concentration (by synthesis and/or uptake) of compatible solutes or osmoprotectants [80]. Nevertheless, it is important to highlight that bacteria do not always activate the same responses or genetic factors when facing osmotic stress produced by different solutes (NaCl, KCl, sucrose, etc.) [81]. For potassium uptake, microorganisms have an inducible high-affinity system (Kdp) and low-affinity systems (Trk, Kup) [82]. On the other hand, the main compatible solutes are glycine-betaine, carnitine, ectoine, proline, and trehalose, and several genetic loci are responsible for their synthesis or uptake [78,79,81]. For example, *L. monocytogenes* at high salt concentrations (10–20%) survives mainly due to the accumulation of glycine-betaine, carnitine, and proline taken up from the environment. The accumulation of glycine-betaine and carnitine occurs via two glycine-betaine transporters which are encoded by the *BetL* gene and the *Gbu* operon. On the other hand, carnitine is internalized via a carnitine transporter encoded by the *OpuC* operon [77]. Some master regulators of the bacterial stress response, such as σB, which are induced by a wide spectrum of stress conditions and which control the expression of numerous genes that mediate the adaptation to suboptimal environments, seem to be involved in the regulation of this complex response to hyperosmotic environments. In fact, for example, both *BetL* and *OpuC* have putative σB-dependent promoters [73].

Some bacteria can display additional survival strategies such as the over-expression of sodium efflux systems, the induction of modifications in cell morphology or membrane fatty acids composition, and the synthesis of specific stress proteins [69,77,79]. In fact, it has been described that cold-shock proteins (Csp), salt-shock proteins (Ssp), and stress-acclimatation proteins (Sap) can contribute to osmotic stress resistance [73].

Additional effects of salt. Sodium chloride exerts its preservative action primarily by making water unavailable for microorganisms and enzymatic reactions, but it also operates a direct antimicrobial effect [47]. High salt concentrations may interfere with the action of several cellular enzymes and force cells to expel Na^+^ ions, which can be very effective in the inhibition of some microorganisms that do not have the necessary tools to counteract those effects. In addition, salt can favor lipid oxidation and thus can affect the quality of meat. Salt (0.5–2%) was found to be pro-oxidant on ground beef and in pork (1.5% salt). The pro-oxidant action of NaCl could be explained because NaCl can disrupt the cell-membrane-liberating ions that form the molecules and finally inhibit the enzymes that are in charge of antioxidant activity [83]. Enzymatic activity can also be affected by low a_w_ values caused by the addition of salt. It was observed that cathepsins, aminopeptidases, and neutral lipases were strongly affected as a_w_ values were lowered from 1 to 0.85 during ham dry curing. Nevertheless, other enzymes like calpain, acid lipase, or acid phospholipase were less or not affected at all [84]. It should also be considered that salt is usually formulated not as pure NaCl but as curing salt (sodium chloride containing around 12% of sodium nitrite) [9,57]. Sodium nitrite has a particular inhibitory effect on some pathogens, such as *C. botulinum*, and helps modulate the microbiota of meat products [85,86].

Consequences for microbial behavior (growth, survival and inactivation). When exposed to high salt concentrations, microbial cells are forced to consume energy for homeostasis, maintenance, and reparation tasks, to the detriment of other energy-demanding cellular processes, such as growth and multiplication [87]. As a rule of thumb, the lag time increases and the growth rate progressively slows down as the conditions in the surrounding environment deviate from the optimal situation [88].

Another consequence is that the susceptibility of microbial cells to other stress factors can be modified [89]. The induction of cross-protection responses by exposure to low a_w_ environments can modulate the fate of pathogenic microorganisms in food products, impacting shelf-life and food safety. Indeed, it has been reported that microbial cells are more resistant to different processing technologies, such as thermal treatments or high hydrostatic pressure (HHP) at low a_w_ conditions [90,91], and that previous exposure to salt or osmotic stress conditions can increase microbial tolerance to them [92,93,94]. In meat processing, the co-existence or succession of two or more stresses is common, and the sequence of events can be an important factor. For example, an acid stress placing a large energy demand on the cell could greatly sensitize the cell to successive treatments, such as a_w_ stress. Certain cellular components such as cold-shock proteins (Csps) have been shown to contribute to resistance to osmotic stress by means of an adaptive response [95]. Therefore, the combined or sequential exposure of cells to two or more stresses in food environments might induce cross-protection responses [96,97].

## 4. Changes in the Microbiota of Meat Products Due to Salt

Fresh meat is particularly prone to microbial spoilage due to the abundance of nutrients and favorable intrinsic properties that do not hamper the metabolism of bacteria. When microbial activity impairs organoleptic properties, such as odor, taste, texture, or appearance, the food is considered unfit for human consumption and rejected [98,99,100].

The manufacture of meat products can be seen as a successful preservation method based on the shifting of microbial populations from spoilage and (sometimes) pathogenic Gram-negative bacteria to desirable and beneficious Gram-positive ones (such as many species of *Lactobacillaceae* and *Micrococcaceae*), which confer attractive sensory properties and achieve long shelf-lives [101]. This shift is attained mainly by processes and factors such as salting/curing, temperature control, atmosphere modification, acidification, drying, or the use of antimicrobials and can be reinforced by the addition of selected starter cultures [60,102,103]. Thus, salt contributes to enlarging the shelf-life of meat products and improving safety because it fosters a shift in the dominant microbial populations originally occurring in the raw material [104].

Salt is able to inhibit the growth of many spoilage and pathogenic bacteria, yeasts, and molds, albeit to a different extent depending on the microbial group [77]. Halophiles have been shown to contain enzymes active in solutions of very high ionic strength [105], while the salt tolerance of non-halophiles is related to their ability to accumulate potassium and other compatible solutes within the cells [106]. In general, Gram-positive bacteria isolated from foods are more tolerant to salt than Gram-negatives.

The inhibitory effect on microorganisms takes place in the aqueous phase of the product. Therefore, data such as salt concentration in the aqueous phase and/or a_w_ are preferable for estimating the inhibitory effect on the microbiota. The majority of spoilage bacteria grow at a_w_ above 0.90, but some can grow at a_w_ as low as 0.85 and in extreme cases even lower [69,91]. Yeasts and molds in general tolerate a lower a_w_ and many can grow at an a_w_ down to about 0.7 (down to 0.6 for some xerophile species) [69]. Among them, some fungi can produce mycotoxins under low a_w_ conditions. Salt is a powerful inhibitor of Gram-negative bacteria, which commonly colonize the surface of aerobically-stored refrigerated fresh meat and degrade low-molecular-weight compounds with the ultimate production of substances that contribute to off flavors and tastes [107,108]. Non-motile aerobic rods and coccobacilli belonging to *Pseudomonas*, *Moraxella*, *Psychrobacter*, *Acinetobacter*, and psychrotrophic *Enterobacteriaceae* are major components of the spoilage microbiota of refrigerated raw meat stored aerobically [107,108]. Fresh meat spoilage is preceded by a time-variable phase in which bacteria use low-molecular-weight compounds such as glucose and glucose-6-P as a carbon and energy source. Later on, Gram-negative bacteria use amino acids at refrigeration temperatures and typical pH conditions in post-mortem meat to obtain energy [109,110], which occurs when high amounts of bacteria (more than 10^7^ CFU/cm^2^) are present.

If environmental conditions change, a selection pressure on the bacterial community is exerted, and certain groups become dominant. The prevailing conditions during ripening (a_w_, pH, Eh, NaCl concentration, etc.) favor the growth of microbial groups such as lactic acid bacteria (LAB), *Micrococcaceae*, certain yeasts, and molds, which dominate the microbiota of meat products [102]. These microbial groups are able to decrease the pH and convert lipids and proteins into desirable substances during the curing process [111,112]. The spoilage capacity of these groups is usually limited, and the end-products of their metabolism are not overtly offensive, although some Gram-positive species can be very detrimental [110]. In the industrial manufacture of some fermented meat products, ad hoc starter cultures from the above-mentioned groups are used to improve the quality and safety of the product by accelerating the change in microbial populations, displacing the spoiling microbiota [112,113,114,115].

### 4.1. Microbiological Safety Assessment and Shelf-Life of Low Salt Meat Products

Reducing the salt content of a particular meat product can represent a challenging task for the industry. The contribution of salt to the technological and sensory quality of the final product can be replaced, but the potential safety risks linked to reformulations should be assessed more carefully [20,53,116]. When microbiological safety is the major issue considered, the straightforward approach is to determine the likelihood of the growth or survival of spoilage and pathogenic microorganisms in the specific low-salt meat product [16,48]. For this purpose, it is necessary to initially identify the hazards and other microbiota occurring in the product; secondly, to evaluate the inhibitory capacity of other intrinsic and extrinsic conditions (hurdles) in the product; and, finally, to consider the technological steps that are feasible and suitable for achieving microbial stability. Predictive models, challenge testing, and shelf-life tests are suitable tools to obtain accurate estimates of the likelihood of the growth, survival, or inactivation of microorganisms in the product [117,118,119]. The implementation of an efficient strategy to guarantee the microbiological safety of low-salt meat products is achievable once all data are gathered and the adequate tools are suitably used.

Most of the scientific literature published on low-salt meat products deals with the sensory and technological issues, although there are also some articles that investigate the microbiological aspects [17,65,120,121]. Numerous articles show that, when NaCl levels used are below those typically formulated in meat products, the shelf-life is significantly reduced [53,104]. For example, the shelf-life of a typical Greek pork meat product (4.85% *w*/*w* NaCl) was reduced by between 10 and 78 days when the salt content was 50% reduced and samples were stored between 15 and 0 °C, respectively. In this research article, Michelakou et al. (2021) evaluated the influence of 50% reduced salt and incubation temperatures (0, 5, 10, and 15 °C) on a pork product. It is important to consider that, at higher incubation temperatures, the incubation period was reduced, both for the control samples and the reduced-salt samples [65]. A salt reduction promoted faster spoilage of raw sausages by lowering the overall bacterial diversity (both richness and evenness) in the product, including Gram-negative as well as Gram-positive bacteria [104]. Although no apparent changes were noticed in shelf-life, both aerobic and LAB counts increased significantly after 60 days of storage in low-salt turkey sausage formulations [122]. The authors concluded that a salt replacer could effectively and completely substitute NaCl in smoked turkey sausages, although some sensorial optimization may be required. The results obtained by Charmpi et al. (2020) suggest that the salt level (between 1 and 4%) influenced the diversity of microbial communities during the fermentation of pork minced meat. The highest salt concentration lowered the bacterial diversity, as Enterobacterales were detrimentally affected. LAB and coagulase-negative staphylococci predominated during the fermentation process, as they are well adapted to higher-salt environments (6% NaCl in regular sausage products) [60,115].

### 4.2. Microbial Hazards Associated with Low-Salt Cured Meat Products (Hazard Identification)

Bacterial pathogens can occur in the meat product because the raw materials are contaminated (meat, offal, spices, and other ingredients) or as a consequence of non-hygienic manipulation, bad manufacturing practices, and cross-contamination from utensils and equipment at the processing facilities. Once they have reached the products, the manufacturing and storage conditions (including temperature-time combinations, a_w_ of the foodstuff, and preservative concentration) dictate whether bacteria can grow, survive, or are inactivated [99,100,123]. If a significant inhibitory barrier, such as the salt, is reduced, the ability of pathogens to survive and grow increases, but the risk is likely greater for those microorganisms more susceptible to salt inhibition. Table 2 shows the tolerance limits for a_w_ of selected bacteria.

The identification of microbiological hazards linked to a product as one of the steps in a HACCP plan is a necessary phase that the industry should complete. The assessment has to consider the prevalence and concentration of biological hazards potentially present in the raw materials or introduced during food handling and processing, the intrinsic and extrinsic conditions during processing, and the conditions of storage and distribution [124]. The safety assurance is improved when, in addition to a proper risk evaluation, procedures for assessing the lethal effect of the treatments are included, as well as mechanisms to monitor, evaluate, optimize, and validate the lethal burden of the process. Epidemiological data from outbreaks linked to meat products [125], expert elicitation, and data from source attribution are all useful in identifying and ranking the main pathogens associated with meat products [126]. The published scientific literature and risk assessments constitute valuable data to carry out a hazard identification, but the most important data are aspects related to the hygienic and manufacturing conditions in a given factory (e.g., hygienic quality of raw materials, intrinsic factors such as fermentation temperature, storage time, etc.) [26].

*Salmonella* has usually been reported as a causative agent in outbreaks and cases of infection linked to the consumption of meat products [127]. The contamination with *Salmonella* of raw products of animal origin (meat, fat, spices, etc.) can occur very frequently and has been reported in many research articles [127,128]. Warm-blooded animals are frequent *Salmonella* reservoirs, and, therefore, *Salmonella* can contaminate the meat during slaughtering and meat processing. Cross-contamination and recontamination events linked to *Salmonella* during meat processing and preparation are also recurrently described in the literature [129,130]. The low a_w_ values achieved during the curing of traditional dry-cured salami or loins have been linked with a reduction in *Salmonella* presence [131,132], although the NaCl content did not significantly affect the probability of finding *Salmonella* [131]. *Salmonella enterica* can survive hyperosmotic stress conditions due to high NaCl concentration (6%), and its survival ability is influenced, as in other Gram-negative foodborne pathogens, by the alternative sigma factor RpoS [133]. Raybaudi-Massilia et al. (2019) did not find any significant differences regarding the occurrence of *Salmonella enteritidis* when comparing control samples from three meat products (cooked ham, 1.14% g Na: turkey breast, 1% g Na: and Deli type sausage, 1.33% g Na) with samples with up to a 30% reduction in salt content [19].

Shiga-toxin-producing *Escherichia coli* (STEC) are zoonotic agents characterized by the production of Shiga-like toxins commonly associated with foodborne disease episodes that can lead to severe health complications and sometimes death. Although the majority of reported STEC cases have been linked to strains of serotype O157, other serotypes, such as O45, O26, O91, O103, O111, O121, and O145, are emerging as causative agents of foodborne disease [127]. In the European Union, 4446 confirmed cases of STEC infections were reported in 2020, with a notification rate of 1.49 cases per 100,000 population. STEC has usually been associated with meat from ruminants, and the main food vehicles implicated as the source of outbreaks are bovine meat and meat products, together with other types of food and water [127]. As with *Salmonella*, *E. coli* is susceptible to low a_w_ values, and the numbers decrease as the curing process advances [134]. A short curing period has been identified as one of the factors responsible for an outbreak attributed to fermented sausages due to STEC [135].

*Listeria monocytogenes* also represents a hazard in meat and meat products. In 2020, 1876 confirmed cases of listeriosis were reported in the EU. It was the zoonosis that had the highest case-fatality rate (13.0%). No statistically significant increasing trend was observed during the 2016–2020 period [127], although a significant increasing trend was observed in previous years (2008–2016) [136]. *L. monocytogenes* is considered salt tolerant [137]. Indeed, it has been reported that growth can occur at NaCl concentrations as high as 10% and even more in the case of adapted strains [73]. Thus, microbial reduction in response to a low a_w_ is less accentuated when compared to other pathogens [92,132]. In sliced chouriço, salt acted as an effective hurdle to control *L. monocytogenes* growth, and manufacturing meat products with lower salt content (1.5% as compared to 3%) allowed the growth of the pathogen [67].

In general, Gram-positive bacteria are able to grow at lower a_w_ conditions compared to Gram-negative bacteria, and *Staphylococcus aureus* is the pathogenic bacteria with the lowest minimum growth a_w_. *S. aureus* can grow in conditions of high salt concentration (10–20%) (a_w_ = 0.83 to 0.86) and performs better than other competitive flora under low a_w_ due to its great adaptative response to osmotic stress [138], although it does not produce enterotoxin in such conditions (it only produces enterotoxin at a_w_ > 0.90). Stress conditions, such as NaCl stress (4.5%), were shown to decrease *seb* (staphylococcal enterotoxin B) promoter activity [139].

In meat products, botulism outbreaks have usually been associated with food processing failures (thermal treatment) and the irregular distribution of curing salts, which allows spore outgrowth and botulism toxin production [140,141]. The products that are often implicated are home-made canned meat products and cured hams with curing defects and anaerobic conditions in the inner parts of the product that allow the germination of *C. botulinum* spores. Curing salts (nitrate and/or nitrite), independently of the salt formulation, have been shown to be adequate preservatives for the control of *C. botulinum* in dry-cured hams salted with formulations including replacers such as KCl and/or CaCl_2_ and MgCl_2_ [85].

A lower level of biogenic amines (particularly cadaverine, histamine and tyramine) has been reported in blood dry-cured sausages and traditional Portuguese sausages manufactured with a level of 3% salt as compared to 6% [115,142].

### 4.3. Processing Intrinsic and Extrinsic Hurdles Affecting Microbial Hazards in Meat Products

Salting, together with other classical food preservation processes (drying, freezing, thermal treatment, etc.), imposes extreme physical and chemical barriers on microbial growth and has traditionally been used in many instances as common methods of preservation (Table 3). For all these preservation processes, microbial stability for long periods of time is achieved using stringent conditions that constitute robust obstacles or “hurdles” for bacteria, even though they dramatically change the organoleptic characteristics of fresh meat. Meat products manufactured in this way are very different from fresh meat in their organoleptic characteristics [143].

In contrast, modern strategies in food preservation seek to apply mild treatments to inactivate or permanently inhibit injured microorganisms by using multiple barriers, especially in the case of minimally processed foods [144]. This is the reaction of the food industry to the demands and preferences of modern consumers in relation to quality, healthiness, nutrition, convenience, and hedonic perception [144,145]. Low-salt meat products are a perfect example, in which sensory attributes and microbial stability should be achieved using a combination of methods or preservation technologies and favorable intrinsic and extrinsic factors. This approach has been visualized as a sequential or simultaneous group of hurdles acting synergistically to inhibit the growth of or inactivate microbes [20,144]. An effective and stable system capable of prolonging the shelf-life and assuring the safety of the end-product is accomplished when the combined hurdles inactivate most of the spoilage and pathogenic microorganisms while survivors are inhibited. Microbial growth and pH remained within the normal range in sausages when the sodium chloride was replaced with 20% potassium chloride and 38% calcium chloride in combination with an olive oil emulsified alginate [27]. Some hurdles can achieve a complete microbial inactivation and thus have a bactericidal effect, (e.g., thermal treatment, HHP, acidification), while others only slow down or arrest the microbial growth (bacteriostatic effect) depending on the intensity of the hurdle (salting, refrigeration, use of modified atmospheres) [146]. As part of the recent methods to be used in hurdle technology, the use of food cultures and/or their metabolites (i.e., bacteriocins) as natural preservatives in food should be highlighted [68,147,148,149,150].

From a safety viewpoint, when meat processing does not include a killing step, the use of cumulative and synergistic hurdles is strictly necessary to maintain the safety and stability of the meat product since the inactivation of pathogens or spoilage agents cannot be completely guaranteed. In general, a number of microorganisms would be able to grow at the NaCl concentrations (<5%) encountered in most meat products in conditions of optimal temperature, pH, and nutrient availability. However, the presence of these additional growth barriers (acidity, refrigeration storage, vacuum or modified atmosphere packaging, the presence of other antimicrobial compounds such as nitrites, preservatives, food cultures, etc.) can slow down or stop microbial metabolism when combined with the relatively mild salt concentrations prevailing in this type of meat product.

The restrictions in the application and intensity of those hurdles come from constraints such as the maintenance of the sensory quality of the product (flavor, texture…), its conformity with legislative requirements (additive maximum limits), and the ability to meet the economic industrial demands (reduced costs, water loss…) [53,146]. Some non-thermal preservation technologies (pulsed electric fields, irradiation, electromagnetic fields, etc.) are not appropriate for the manufacture of meat products for technological reasons or limited consumer acceptance. On the other hand, technologies such as HHP are very suitable for the manufacture of low-salt meat products due to their capacity to inactivate the microbiota while contributing to protein solubilization [20,151,152,153,154,155,156].

**Table 3:** Use of cumulative and synergistic hurdles with salt to achieve the safety and quality of meat products.

Product	Combined Hurdles	Results	Reference
Raw pork meat	350 MPa HPP + 1, 1.5 or 3% NaCl	Synergism between HPP and salt showed to control bacteria recovery (aerobic mesophiles, LAB and *Enterobacteriaceae*) more than each hurdle alone during storage.	[156]
Sliced dry cured ham	2.8% NaCl + 600 MPa	Combined hurdles achieved *Salmonella* and *L. monocytogenes* inactivation 14 and 42 days earlier than HPP alone.	[157]
Sausage (*chorizo*)	1.01% NaCl + 0.48% KCl + 0.91% CaCl_2_ + olive oil emulsified alginate replacing pork fat	A reduction of 58% NaCl in sausages seems to be feasible since no pH and microbial counts remained in the normal values.	[27]
Pork sausage	600 Mpa HPP + carrot fibers or potato starch + 1.2% NaCl	Reducing salt content from 1.8% to 1.2% with the addition of HPP and hydrocolloids did not negatively influence the water binding capacity, color, or texture of sausages.	[154]
Sheep natural sausage casings	0, 4, 7 or 12% NaCl + 0, 100, 150, 200 ug/g nisin	Combined hurdles greatly controlled *L. monocytogenes* than salt and nisin alone.	[158]
Pork	Ultrasound (9 and 54.9 W/cm^2^) + 5% NaCl or a commercial salt replacer	Ultrasound only enhanced NaCl diffusion into the meat but did not influence the replacer diffusion.	[159]

## 5. Strategies to Guarantee the Microbiological Safety of Low-Salt Meat Products

Several strategies have been devised to guarantee the microbiological safety of low-salt meat products (salt replacers, antimicrobial compounds, flavor enhancers, improved salt application techniques, processing technologies) [20]. The strategies can be classified using different approaches (Table 4).

First approach is to replace NaCl (totally or partially) using other additive(s) with similar properties [38,61,143,160]. A 20% sodium reduction was obtained in turkey breast by replacing NaCl with Na_2_HPO_4_ prepared in a 50:50 blend [161]. The replacement needs to be carefully adjusted since the inhibitory barrier of the substitute may be lower than that of NaCl [162,163,164]. In addition, there is a synergistic effect of NaCl with other hurdles, which may be absent with the replacer [165]. The reduction of the sodium content (by reducing both salt and sodium nitrite) allows a rapid growth of lactic acid bacteria and proteolytic microorganisms in cured meats, resulting in a product that spoils more rapidly [48]. Dry fermented sausages with a 58% NaCl substitution with KCl and CaCl_2_ showed a more pronounced growth of *Lactobacillus* than the control sample (2.4% NaCl). Moreover, *Lactobacillus* counts in the control decreased (2–3 log cfu/g) during ripening, while they maintained more or less stable levels in reduced sausages [27]. NaCl is very effective in controlling pathogens and spoilage organisms, thus it can be necessary to substitute its inhibitory action by using some other preservatives in case of replacement [121]. A higher amount of yeast (4.7–5.4 log cfu/g) was found in a 10% salt content bacon, while lower counts (1.3–3.9 log cfu/g) were found in 1.4% salt content bacon, which might indicate that a higher salt content is expected to suppress the growth of bacteria, enabling the slower-growing yeast to better grow in the product [16]. Alternative salts (e.g., KCl, MgCl_2_, CaCl_2_, MgSO_4_, etc.), sugars, proteins, and humectants decrease the a_w_ in foods, but they usually have an inferior inhibitory action. Replacers of salt and other sodium-containing preservatives, such as KCl [20], mixtures of potassium lactate and sodium diacetate [166], or mixtures of KCl, MgCl_2_, and CaCl_2_ [167,168] are not as effective in the inhibition of undesirable microbiota. Other a_w_ depressors have no inhibitory effect at all. The replacement should also consider the other functions of salt (sensory, technological role, flavoring agent) in the meat product [9,143,169,170,171,172]. In a mortadella product, lower sensory acceptability, especially regarding flavor, was found when blends containing 1% NaCl, 0.5% KCl, and 0.5% MgCl_2_ and 0.5% NaCl, 1% KCl, and MgCl_2_ 0.5% were used in the formulation compared to the 2% NaCl control [173].

Another strategy is to increase the intensity of the remaining (intrinsic and extrinsic) hurdles, so they compensate for the reduction of salt and its inhibitory potential. Examples of these meat products are commercially-sterile canned products, products with a pH under 3.8 (submitted to an intense acidification), frozen products intended for immediate consumption after thawing, products with a low a_w_ achieved by other means (e.g., extensive drying that increases concentrations of solutes in the final product), and natural seasonings [174]. García-Lomillo et al. (2017) achieved a 1% salt reduction when using 2% red wine pomace seasoning. However, these types of products may present a strong or defective sensory profile, due to an extreme application of one single barrier.

One more procedure is to include a further processing step, i.e., a decontamination treatment, applied either to the raw materials before processing or to the final meat product once it has been manufactured and packaged [8]. The introduction of an inactivation step for raw materials (thermal treatment, HHP, light pulses, chemical decontamination, etc.) may be difficult to put into practice, due to unwanted modifications that occur in fresh meat and sensory changes in the final product [175]. The second option (treatment of the final product) is very effective since the process’s safety assurance is enhanced, as recontamination is prevented [176,177,178]. In any case, the introduction of an inactivation step in the food-manufacturing process requires a careful assessment of microbiological risks, including an adequate calculation of the lethality effect. There is also a need to have tools and instruments to monitor, optimize, and validate the process on-line and procedures to model the lethal effect of the treatment [179,180,181]. A combination of this option and a replacer or other additional hurdles in the formulation have also been proposed, with HHP as the most favored choice [20,155,182].

A final option is to use raw materials of optimal microbiological quality in the manufacture of low-salt meat products by increasing the hygienic standards at the slaughterhouse and cutting plant and strictly adhering to HACCP and GMP (good manufacturing practices), including environmental monitoring and sanitation. On its own, this procedure is considered insufficient to produce stable low-salt meat products and should be complemented by other methods, such as those listed above. In any circumstance, it is always necessary that the processing of meat products should adhere in all circumstances to the strictest conditions of process hygiene [175].

Either way, the safety of the whole process (formulation, hurdle combination, and processing steps) should preferably be verified by challenge testing and aided by mathematical modelling.

## 6. Use of Challenge Testing and Shelf-Life Tests

Challenge testing and shelf-life tests are useful tools that help food processors determine the quality of foods and estimate the ability of foodborne pathogens to grow during the foreseeable conditions of distribution and storage. This is especially necessary when changes in the product formulation (e.g., lowering salt content) are introduced, as the possibility of reformulated low-salt meat products having shorter shelf-life or causing foodborne illness has already been emphasized. Salt replacement or reduction has an impact on the a_w_ of the food and, therefore, will undoubtedly modify the growth behavior of pathogenic and spoilage microorganisms; therefore, there is a need for effective tools ensuring the manufacture of safe foods with changes in the shelf-life [62,183].

The microbial growth ability in food products can be estimated based on specifications of the physico–chemical characteristics of the product, consultation of the available scientific literature, or predictive mathematical modelling (see below). In many cases, a growth assessment will have to involve laboratory-based studies, so-called challenge tests, and shelf-life studies [75]. Challenge and shelf-life testing is normally performed on a case-by case basis, which means that it can be very expensive and time-consuming, particularly if a range of products, formulations, and different bacteria have to be tested. Results can take many days until they are available, since they are usually obtained by classical microbiological analysis. Nonetheless, both tests can provide valuable information on microbial stability to food processors.

Challenge testing. As a primary objective, challenge tests aim to determine whether a particular food product has the ability to support the growth of a particular microorganism. Simulation of conditions in an artificially contaminated product allows us to study the fate of pathogens or spoilage microbiota. In any case, results should be analyzed with care (including fail-safe approach), considering all the constraints and assumptions introduced in simulating the natural contamination present in foods and the accurate reproduction of conditions of foods during storage, distribution, sale, and preparation. Challenge testing is a technique commonly employed in research [62,184,185,186,187,188]. Up to a 40% NaCl reduction was achieved during a challenge test in a pre-packed cooked meat product when it was replaced with a commercial mixture of potassium lactate and sodium diacetate without statistically affecting the shelf-life [166]. In a challenge test carried out in salami with 4% NaCl and 2.8% NaCl plus 1.6% potassium lactate, the reduced and replaced sample showed to be effective with respect to microbial benefits without compromising the product quality [62]. According to the authors, a limitation of this challenge test could be the absence of exposure to abusive temperatures, which does not allow the interpretation to be further extended to other storage temperatures.

Shelf-life studies. In the European Union, Regulation (EC) No 2073/2005 allows food-business operators to carry out shelf-life studies, as necessary, to investigate compliance with the food-safety criteria throughout the product’s lifespan. They are conducted to study whether particular microorganisms are able to survive and grow in naturally contaminated foods during storage and distribution beyond the limits imposed by the Regulation. The consultation of the available scientific literature and specifications of physico-chemical characteristics of the product is encouraged. For example, referring to *L. monocytogenes*, according to the EURL Lm technical guidance document for conducting shelf-life studies on *Listeria monocytogenes* in ready-to-eat foods [183], shelf-life tests for *L. monocytogenes* would not be needed for the following meat products [189]:

- foods produced for immediate consumption (with a shelf-life of less than five days);
- foods (meat products) which are intended to be cooked or subjected to any other bacterial inactivation step before human consumption;
- foods which have received a heat treatment or other processing effective to eliminate *L. monocytogenes*, when recontamination is not possible after this treatment (e.g., products treated in their final package);
- meat products with pH ≤ 4.4, or a_w_ ≤ 0.92, or pH ≤ 5.0 and a_w_ ≤ 0.94, conditions which are already known as unable to support the growth of *L. monocytogenes*; and
- other categories of product can also belong to this category, subject to scientific justification (e.g., frozen products).

Moreover, historical data on the prevalence of the particular microbial species in the specific food product at the end of its shelf-life and particularly on results of durability studies (the number of samples exceeding 100 CFU/g) and outputs of predictive microbiology modules may be useful in deciding whether a test is required or not for a particular foodstuff.

Meat products contain several hurdles that impose a series of restrictions affecting the microbial growth potential of a given pathogen (see above). This growth potential can serve to classify foods or to evaluate particular food products with regards to shelf-life and safety. When the growth potential is lower than 0.5, it is considered that the intrinsic and extrinsic properties of the product are able to restrict pathogen growth during shelf-life, in case an accidental contamination of the product has taken place. Nonetheless, this aspect does not eliminate the risk or probability of diseases associated with these products, as the sole presence of the pathogen in the product implies a certain degree of exposure.

## 7. Predictive Microbiology in the Safety Assessment of Low-Salt Meat Products

Predictive microbiology uses mathematical functions to describe the behavior of microorganisms subjected to intrinsic and extrinsic factors in foods. For this purpose, diverse software tools (ComBase, Monte Carlo simulation, Decision Tools @Risk, MicroHibro, etc.) are available that allow to users calculate the growth, survival, and inactivation of bacteria in foods. Models attempt to estimate the quantitative or qualitative evolution of microbial populations over time and, therefore, the food shelf-life and pathogen fate.

Models that describe the growth of a population of microorganisms are being increasingly used to adopt strategies to improve food safety. From such a point of view, models have to be able to calculate and describe the growth, survival, or inactivation of spoilage or pathogenic bacteria in the food matrix under a defined set of extrinsic and intrinsic conditions and, eventually, when microbial numbers might reach a level compromising human health [117]. A variety of deterministic models describing the bacterial growth, survival, and inactivation in meats in response to environmental factors (temperature, pH, water activity, etc.) have been proposed [92,190,191]. Models are based on variations of the Bigelow, Baranyi, Gompertz, Logistic, or Richards models, with the environmental effects being expressed through changes in the equation parameters (Lopez et al., 2004). In addition, some models have also been published describing the fate of pathogens (growth, inactivation, and survival) under (static or dynamic) conditions of processing, studying the impact of extrinsic and intrinsic factors on meat products [92,179,180,181,192,193,194,195]. A model to describe the combined effect of salt and heating temperature on the heat tolerance of *L. monocytogenes* was described for meat and seafood products to achieve ≥3 log_10_ reductions. Only the products with salt influenced the model, thus being independent from strains, temperatures, and type of food [196]. Nevertheless, the authors are aware that other intrinsic factors might influence the model, and that it needs deeper research. In another modelling study, *L. monocytogenes* growth was stimulated at 0.92–0.94 a_w_ when 4% NaCl was applied [197].

New genetic, physiological, and molecular information is increasingly available, which will improve the prediction capacity of models. In any case, the use of this methodology requires a high level of expertise [198]. Assumptions and limitations should be taken into account, e.g., information available is often obtained from studies carried out under optimal conditions (37 °C, neutral pH, etc.) and in laboratory-based rich media.

Using software with growth/no growth boundary modules, it is possible to obtain information on the growth probability of pathogens according to pH, a_w_, and temperature. The models that investigate the growth–no growth interface of target microorganisms are particularly useful for these purposes, since they can afford information on the impact of intrinsic and extrinsic factors to determine the behavior of pathogens or spoilage microorganisms in the final product. Similarly to other processes, the validation of mathematical models in foods is necessary, together with challenge tests and shelf-life tests, especially if the assessments are performed in model systems.

## 8. Conclusions

Most of the scientific literature published on low-salt meat products deals with sensory and technological issues, while the safety viewpoint has been somehow overlooked. There is a need to further investigate the microbiological implications of salt reduction in meat products, since the inhibitory barrier offered by salt may not be adequately replaced. The assessment of the safety risk associated with meat products with a low salt concentration should be unavoidably performed on a case-by-case basis. To achieve this aim, mathematical modelling, challenge tests, and shelf-life studies are very useful tools that should be used by experienced personnel. In the industry setting, all this information should be assessed and integrated into an HACCP plan that includes a comprehensive hazard-identification phase and adequate tools able to control and monitor the critical points. To guarantee the microbiological safety of low-salt meat products, approaches can be addressed towards finding suitable replacers (salts or other depressors of a_w_), processing changes that increase the intensity of remaining hurdles (intrinsic and extrinsic factors), the use of processing technologies able to decontaminate the end product, the use of more than one strategy as a part of hurdle technology (HPP, use of food cultures and/or natural antimicrobial compounds, etc.), or the (always) necessary hygienic strategies able to obtain raw materials with a low amount of microbial contaminants. The reduction or elimination of salt associated with a product reformulation has to ensure the same safety level, must be economically and technologically viable, and must be accepted by the consumer from a sensory point of view.

## Figures and Tables

**Table 1:** Salt content in a selection of meat products from different countries.

Product	NaCl Content	Country	Reference
Beef, cured, dried beef	8.68	USA	[54]
Pork, cured, bacon, cooked, broiled, pan-fried, or roasted	3.99	USA	
Pork sausage	3.23	USA	
Canned meat chop	3.44	Serbia	[55]
Cooked sausages	2.95	Serbia	
Smoked products	3.44	Serbia	
Hard pork sausage	3.18	Spain	[56]
Cooked ham	2.45	Czech Republic	[57]
Frankfurters	2.44	Czech Republic	
Knackauer	2.34	Germany	
Schinkenwurst	2.03	Germany	
Bierschinken	2.2	Germany	
Pancetta	2.94	Serbia	[58]
Kulen sausage	4.24	Serbia	
Chorizo	3.58	Spain	[52]
Fuet-type sausage	3.94	Spain	
Mortadella sausage	1.97	Spain	

**Table 2:** Limiting conditions regarding a_w_ for the growth of bacterial pathogens (adapted from Table A-1 in (FDA, 2011) [71]).

Bacterial Pathogens	Min. a_w_	Max. % Water-Phase NaCl	Min pH
*Bacillus cereus*	0.92	10	4.3
*Campylobacter jejuni*	0.987	1.7	4.9
*Clostridium botulinum*, type A, and proteolytic types B and F	0.935	10	4.6
*Clostridium botulinum*, type E, and nonproteolytic types B and F	0.97	5	5.0
*Clostridium perfringens*	0.93	7	5
*Escherichia coli*	0.95	6.5	4
*Listeria monocytogenes*	0.92	10	4.4
*Salmonella* spp.	0.94	8	3.7
*Shigella* spp.	0.96	5.2	4.8
*Staphylococcus aureus* growth	0.83	20	4
*Staphylococcus aureus* toxin formation	0.85	10	4
*Vibrio cholerae*	0.97	6	5
*Vibrio parahaemolyticus*	0.94	10	4.8
*Vibrio vulnificus*	0.96	5	5
*Yersinia enterocolitica*	0.945	7	4.2

**Table 4:** Approaches to guarantee the microbiological safety of low-salt meat products.

Approach	Main Mechanism	Advantages *	Disadvantages
Use of preservatives that supplement or replace inhibitory power of salt	Low a_w_ and inhibition power of preservatives (KCl, MgCl_2_, CaCl_2_, MgSO_4_, food cultures, bacteriocins, etc.)	Characteristics of the product remain (almost) unchanged.	Need to evaluate inhibitory power. Synergy with other hurdles absent. Sensory and technological properties of replacer should be assessed. No green label.
Increase intensity of remaining hurdles	Stricter conditions that inhibit microbiota (acidification, drying, freezing)	Green label. No need to change formulation, processing equipment.	Products of quite different sensory quality. Economic constraints (e.g., water loss).
Processing technologies. Decontamination	Inactivation of microbiota (HHP)	Avoids recontamination (product packaged). Green label. Useful also for technological properties.	No application to raw materials. Efficacy depends on the characteristics of product. High initial investment.
High level of hygiene in production	Raw materials of good quality with low numbers of spoilage and absence of pathogenic microorganisms (Hygiene, HACCP, GMP)	Strategy that it is beneficial and needed in any event.	Insufficient on its own, needs supplementation with other strategies. Depends on the raw material supplier.

* In addition to those linked to health due to salt reduction.

## Data Availability

Not applicable.

## References

[1] Halagarda M., Wójciak K.M. (2022). Health and safety aspects of traditional European meat products. A review. Meat Sci..

[2] Vidal V.A.S., Lorenzo J.M., Munekata P.E.S., Pollonio M.A.R. (2021). Challenges to reduce or replace NaCl by chloride salts in meat products made from whole pieces—A review. Crit. Rev. Food Sci. Nutr..

[3] Vidal V.A.S., Paglarini C.S., Lorenzo J.M., Munekata P.E.S., Pollonio M.A.R. (2021). Salted Meat Products: Nutritional Characteristics, Processing and Strategies for Sodium Reduction. Food Rev. Int..

[4] O’Donnell M., Mente A., Yusuf S. (2015). Sodium Intake and Cardiovascular Health. Circ. Res..

[5] Coombs C.E., Holman B.W., Friend M.A., Hopkins D.L. (2017). Long-term red meat preservation using chilled and frozen storage combinations: A review. Meat Sci..

[6] Gaudette N.J., Pietrasik Z., Johnston S.P. (2019). Application of taste contrast to enhance the saltiness of reduced sodium beef patties. LWT.

[7] Pietrasik Z., Gaudette N. (2014). The impact of salt replacers and flavor enhancer on the processing characteristics and consumer acceptance of restructured cooked hams. Meat Sci..

[8] Inguglia E.S., Zhang Z., Tiwari B.K., Kerry J.P., Burgess C. (2017). Salt reduction strategies in processed meat products—A review. Trends Food Sci. Technol..

[9] Cruz-Romero M.C., O’Flynn C.C., Troy D., Mullen A.M., Kerry J.P. (2021). The Use of Potassium Chloride and Tapioca Starch to Enhance the Flavour and Texture of Phosphate- and Sodium-Reduced Low Fat Breakfast Sausages Manufactured Using High Pressure-Treated Meat. Foods.

[10] Kromhout D., Spaaij C.J.K., de Goede J., Weggemans R.M. (2016). The 2015 Dutch food-based dietary guidelines. Eur. J. Clin. Nutr..

[11] WHO (2013). Mapping Salt Reduction Initiatives in the WHO European Region.

[12] Palou A., Badiola J.J., Anadón A., Bosch A., Cacho J.F., Cameán A.M. (2010). Informe Del Comité Científico de La Agencia Española de Seguridad Alimentaria y Nutrición (AESAN) En Relación Al Efecto de La Reducción de La Sal En La Seguridad Microbiológica de Los Productos Cárnicos Curados. Rev. Del Com. Científico La AESAN.

[13] WHO (2012). Sodium Intake for Adults and Children. Guideline: Sodium Intake for Adults and Children.

[14] Van Gunst A., Roodenburg A.J.C., Steenhuis I.H.M. (2018). Reformulation as an Integrated Approach of Four Disciplines: A Qualitative Study with Food Companies. Foods.

[15] Food Safety Authority of Ireland (2016). Salt and Health: Review of the Scientific Evidence and Recommendations for Public Policy in Ireland (Revision 1).

[16] Aaslyng M.D., Vestergaard C., Koch A.G. (2014). The effect of salt reduction on sensory quality and microbial growth in hotdog sausages, bacon, ham and salami. Meat Sci..

[17] Fraqueza M.J., Laranjo M., Elias M., Patarata L. (2021). Microbiological hazards associated with salt and nitrite reduction in cured meat products: Control strategies based on antimicrobial effect of natural ingredients and protective microbiota. Curr. Opin. Food Sci..

[18] Zhang X., Yang J., Gao H., Zhao Y., Wang J., Wang S. (2020). Substituting sodium by various metal ions affects the cathepsins activity and proteolysis in dry-cured pork butts. Meat Sci..

[19] Raybaudi-Massilia R., Mosqueda-Melgar J., Rosales-Oballos Y., de Petricone R.C., Frágenas N., Zambrano-Durán A., Sayago K., Lara M., Urbina G. (2019). New alternative to reduce sodium chloride in meat products: Sensory and microbiological evaluation. LWT.

[20] Tamm A., Bolumar T., Bajovic B., Toepfl S. (2016). Salt (NaCl) reduction in cooked ham by a combined approach of high pressure treatment and the salt replacer KCl. Innov. Food Sci. Emerg. Technol..

[21] Stelmasiak A., Damaziak K., Wierzbicka A. (2021). The Impact of Low Salt Level and Addition of Selected Herb Extracts on the Quality of Smoked Pork. Anim. Sci. Pap. Rep..

[22] Kehlet U., Pagter M., Aaslyng M.D., Raben A. (2017). Meatballs with 3% and 6% dietary fibre from rye bran or pea fibre—Effects on sensory quality and subjective appetite sensations. Meat Sci..

[23] Thao T.T.P., Thoa L.T.K., Ngoc L.M.T., Lan T.T.P., Phuong T.V., Truong H.T.H., Khoo K.S., Manickam S., Hoa T.T., Tram N.D.Q. (2021). Characterization halotolerant lactic acid bacteria *Pediococcus pentosaceus* HN10 and in vivo evaluation for bacterial pathogens inhibition. Chem. Eng. Process. Process Intensif..

[24] Lee Y., Cho Y., Kim E., Kim H.-J., Kim H.-Y. (2018). Identification of lactic acid bacteria in galchi- and myeolchi-jeotgalby 16S rRNA sequencing, MALDI TOF mass spectrometry, and PCR-DGGE. J. Microbiol. Biotechnol..

[25] Mani A. (2018). Food Preservation by Fermentation and Fermented Food Products. Int. J. Acad. Res. Dev..

[26] Holck A.L., Axelsson L., Rode T.M., Høy M., Måge I., Alvseike O., L’Abée-Lund T.M., Omer M.K., Granum P.E., Heir E. (2011). Reduction of verotoxigenic *Escherichia coli* in production of fermented sausages. Meat Sci..

[27] Beriain M., Gómez I., Petri E., Insausti K., Sarriés M. (2011). The effects of olive oil emulsified alginate on the physico-chemical, sensory, microbial, and fatty acid profiles of low-salt, inulin-enriched sausages. Meat Sci..

[28] Wyness L.A., Butriss J.L., Stanner S.A. (2012). Reducing the population’s sodium intake: The UK Food Standards Agency’s salt reduction programme. Public Health Nutr..

[29] Trieu K., Neal B., Hawkes C., Dunford E., Campbell N.R.C., Rodriguez-Fernandez R., Legetic B., McLaren L., Barberio A., Webster J. (2015). Salt Reduction Initiatives around the World—A Systematic Review of Progress towards the Global Target. PLoS ONE.

[30] Graudal N.A., Hubeck-Graudal T., Jürgens G. (2016). Reduced Dietary Sodium Intake Increases Heart Rate. A Meta-Analysis of 63 Randomized Controlled Trials Including 72 Study Populations. Front. Physiol..

[31] Aburto N.J., Ziolkovska A., Hooper L., Elliott P., Cappuccio F.P., Meerpohl J.J. (2013). Effect of lower sodium intake on health: Systematic review and meta-analyses. BMJ.

[32] Poggio R., Gutierrez L., Matta M.G., Elorriaga N., Irazola V., Rubinstein A. (2015). Daily sodium consumption and CVD mortality in the general population: Systematic review and meta-analysis of prospective studies. Public Health Nutr..

[33] Mente A., O’Donnell M., Rangarajan S., Dagenais G., Lear S., McQueen M., Diaz R., Avezum A., Lopez-Jaramillo P., Lanas F. (2016). Associations of urinary sodium excretion with cardiovascular events in individuals with and without hypertension: A pooled analysis of data from four studies. Lancet.

[34] Guallar-Castillón P., Muñoz-Pareja M., Aguilera M.T., León-Muñoz L.M., Rodríguez-Artalejo F. (2013). Food sources of sodium, saturated fat and added sugar in the Spanish hypertensive and diabetic population. Atherosclerosis.

[35] Cook N.R., Appel L.J., Whelton P.K. (2016). Sodium Intake and All-Cause Mortality Over 20 Years in the Trials of Hypertension Prevention. J. Am. Coll. Cardiol..

[36] Sterns R.H. (2015). Disorders of Plasma Sodium—Causes, Consequences, and Correction. N. Engl. J. Med..

[37] Bigiani A. (2016). Electrophysiology of Sodium Receptors in Taste Cells. J. Biomed. Sci. Eng..

[38] Pateiro M., Munekata P.E.S., Cittadini A., Domínguez R., Lorenzo J.M. (2021). Metallic-based salt substitutes to reduce sodium content in meat products. Curr. Opin. Food Sci..

[39] Huffman M.D., Lloyd-Jones D.M. (2017). Global Burden of Raised Blood Pressure: Coming Into Focus. JAMA.

[40] Coxson P.G., Cook N.R., Joffres M., Hong Y., Orenstein D., Schmidt S.M., Bibbins-Domingo K. (2013). Mortality Benefits from US Population-Wide Reduction in Sodium Consumption: Projections from 3 Modeling Approaches. Hypertension.

[41] European Commision Salt Campaign. https://ec.europa.eu/health/other-pages/basic-page/salt-campaign_en.

[42] Kloss L., Meyer J.D., Graeve L., Vetter W. (2015). Sodium intake and its reduction by food reformulation in the European Union—A review. NFS J..

[43] Hendriksen M.A.H., Van Raaij J.M.A., Geleijnse J.M., Breda J., Boshuizen H.C. (2015). Health Gain by Salt Reduction in Europe: A Modelling Study. PLoS ONE.

[44] Tsugane S. (2021). Why has Japan become the world’s most long-lived country: Insights from a food and nutrition perspective. Eur. J. Clin. Nutr..

[45] Verma A.K., Banerjee R. (2011). Low-Sodium Meat Products: Retaining Salty Taste for Sweet Health. Crit. Rev. Food Sci. Nutr..

[46] Balestra F., Petracci M., Charis M. (2018). Technofunctional Ingredients for Meat Products: Current Challenges. Sustainable Meat Production and Processing.

[47] Zhang Y., Guo X., Peng Z., Jamali M.A. (2022). A review of recent progress in reducing NaCl content in meat and fish products using basic amino acids. Trends Food Sci. Technol..

[48] Stringer S., Pin C. (2005). Microbial Risks Associated with Salt Reduction in Certain Foods and Alternative Options for Preservation.

[49] Tobin B.D., O’Sullivan M.G., Hamill R.M., Kerry J.P. (2013). The impact of salt and fat level variation on the physiochemical properties and sensory quality of pork breakfast sausages. Meat Sci..

[50] Kim T.-K., Yong H.-I., Jung S., Kim H.-W., Choi Y.-S. (2021). Technologies for the Production of Meat Products with a Low Sodium Chloride Content and Improved Quality Characteristics—A Review. Foods.

[51] Weiss J., Gibis M., Schuh V., Salminen H. (2010). Advances in ingredient and processing systems for meat and meat products. Meat Sci..

[52] Spanish Agency for Consumer Affairs Food Safety and Nutrition (2012). Salt Content of Foods in Spain.

[53] Desmond E. (2006). Reducing salt: A challenge for the meat industry. Meat Sci..

[54] The University of Maine Sodium Content of Your Food. https://extension.umaine.edu/publications/wp-content/uploads/sites/52/2021/01/4059_updated2021.pdf.

[55] Nadežda P., Milica Ž.-B., Željko M., Sandra J., Stojanov I. (2013). Sodium Chloride Content in Meat Products. Arh. Vet. Med..

[56] Perez-Palacios T., Salas A., Muñoz A., Ocaña E.-R., Antequera T. (2022). Sodium chloride determination in meat products: Comparison of the official titration-based method with atomic absorption spectrometry. J. Food Compos. Anal..

[57] Kameník J., Saláková A., Vyskočilová V., Pechová A., Haruštiaková D. (2017). Salt, sodium chloride or sodium? Content and relationship with chemical, instrumental and sensory attributes in cooked meat products. Meat Sci..

[58] Stamenic T., Petricevic M., Samolovac L., Sobajic S., Cekic B., Gogic M., Zivkovic V. (2021). Contents of sodium-chloride in various groups of locally manufactured meat. Biotechnol. Anim. Husb..

[59] Rai A.K., Tamang J.P. (2017). Prevalence of *Staphylococcus* and *Micrococcus* in Traditionally Prepared Meat Products. J. Sci. Ind. Res..

[60] Charmpi C., Van der Veken D., Van Reckem E., De Vuyst L., Leroy F. (2020). Raw meat quality and salt levels affect the bacterial species diversity and community dynamics during the fermentation of pork mince. Food Microbiol..

[61] Kumar S., Yadav S., Pal A. (2021). Studies on the Impact of Partial Replacement of Sodium Chloride with Potassium Lactate on Quality Attributes of Buffalo Calf Meat Rolls. Asian J. Dairy Food Res..

[62] Muchaamba F., Stoffers H., Blase R., Von Ah U., Tasara T. (2021). Potassium Lactate as a Strategy for Sodium Content Reduction without Compromising Salt-Associated Antimicrobial Activity in Salami. Foods.

[63] Yadav B., Spinelli A.C., Govindan B.N., Tsui Y.Y., McMullen L.M., Roopesh M. (2019). Cold plasma treatment of ready-to-eat ham: Influence of process conditions and storage on inactivation of *Listeria innocua*. Food Res. Int..

[64] Ramaroson M., Guillou S., Rossero A., Rezé S., Anthoine V., Moriceau N., Martin J.-L., Duranton F., Zagorec M. (2018). Selection procedure of bioprotective cultures for their combined use with High Pressure Processing to control spore-forming bacteria in cooked ham. Int. J. Food Microbiol..

[65] Michelakou E., Giaouris E., Doultsos D., Nasopoulou C., Skandamis P. (2021). Εvaluation of the microbial stability and shelf life of 50% NaCl-reduced traditional Greek pork meat product “Syglino of Monemvasia” stored under vacuum at different temperatures. Heliyon.

[66] Da Costa R.J., Voloski F.L.S., Mondadori R.G., Duval E.H., Fiorentini M. (2019). Preservation of Meat Products with Bacteriocins Produced by Lactic Acid Bacteria Isolated from Meat. J. Food Qual..

[67] García-Díez J., Patarata L. (2017). Influence of salt level, starter culture, fermentable carbohydrates, and temperature on the behaviour of *L. monocytogenes* in sliced chouriço during storage. Acta Aliment..

[68] Barcenilla C., Ducic M., López M., Prieto M., Álvarez-Ordóñez A. (2022). Application of lactic acid bacteria for the biopreservation of meat products: A systematic review. Meat Sci..

[69] Esbelin J., Santos T., Hébraud M. (2018). Desiccation: An environmental and food industry stress that bacteria commonly face. Food Microbiol..

[70] Cheung P.C.K., Mehta B.M. (2015). Handbook of Food Chemistry.

[71] FDA (2011). Fish and Fishery Products Hazards and Controls Guidance.

[72] Wood J.M. (2015). Bacterial responses to osmotic challenges. J. Gen. Physiol..

[73] Thakur M., Asrani R.K., Patial V. (2018). Listeria monocytogenes: A Food-Borne Pathogen. Foodborne Dis..

[74] Li F., Xiong X.-S., Yang Y.-Y., Wang J.-J., Wang M.-M., Tang J.-W., Liu Q.-H., Wang L., Gu B. (2021). Effects of NaCl Concentrations on Growth Patterns, Phenotypes Associated with Virulence, and Energy Metabolism in *Escherichia coli* BW25113. Front. Microbiol..

[75] Alvarez-Ordóñez A., Broussolle V., Colin P., Nguyen-The C., Prieto M. (2015). The adaptive response of bacterial food-borne pathogens in the environment, host and food: Implications for food safety. Int. J. Food Microbiol..

[76] Begley M., Hill C. (2015). Stress Adaptation in Foodborne Pathogens. Annu. Rev. Food Sci. Technol..

[77] Burgess C.M., Gianotti A., Gruzdev N., Holah J., Knøchel S., Lehner A., Margas E., Esser S.S., Sela Saldinger S., Tresse O. (2016). The response of foodborne pathogens to osmotic and desiccation stresses in the food chain. Int. J. Food Microbiol..

[78] Zeidler S., Müller V. (2019). Coping with Low Water Activities and Osmotic Stress in *Acinetobacter baumannii*: Significance, Current Status and Perspectives. Environ. Microbiol..

[79] Bremer E., Krämer R. (2019). Responses of Microorganisms to Osmotic Stress. Annu. Rev. Microbiol..

[80] Booth I.R. (2014). Bacterial mechanosensitive channels: Progress towards an understanding of their roles in cell physiology. Curr. Opin. Microbiol..

[81] Schuster C.F., Wiedemann D.M., Kirsebom F.C.M., Santiago M., Walker S., Gründling A. (2020). High-throughput Transposon Sequencing Highlights the Cell Wall as an Important Barrier for Osmotic Stress in Methicillin Resistant *Staphylococcus aureus* and Underlines a Tailored Response to Different Osmotic Stressors. Mol. Microbiol..

[82] Liu Y., Ho K.K., Su J., Gong H., Chang A.C., Lu S. (2013). Potassium transport of *Salmonella* is important for type III secretion and pathogenesis. Microbiology.

[83] Mariutti L.R., Bragagnolo N. (2017). Influence of salt on lipid oxidation in meat and seafood products: A review. Food Res. Int..

[84] Toldrá F. (2006). The role of muscle enzymes in dry-cured meat products with different drying conditions. Trends Food Sci. Technol..

[85] Armenteros M., Aristoy M.-C., Toldrá F. (2012). Evolution of nitrate and nitrite during the processing of dry-cured ham with partial replacement of NaCl by other chloride salts. Meat Sci..

[86] Flores M., Toldrá F. (2020). Chemistry, safety, and regulatory considerations in the use of nitrite and nitrate from natural origin in meat products—Invited review. Meat Sci..

[87] Kempes C.P., van Bodegom P., Wolpert D., Libby E., Amend J., Hoehler T. (2017). Drivers of Bacterial Maintenance and Minimal Energy Requirements. Front. Microbiol..

[88] Freitag A., Cluff M., Hitzeroth A.C., van Wyngaardt L., Hugo A., Hugo C.J. (2021). Community level physiological profiling of reduced or replaced salt fresh sausage inoculated with *Escherichia coli* ATCC 25922. LWT.

[89] Shah M., Bergholz T. (2020). Variation in growth and evaluation of cross-protection in *Listeria monocytogenes* under salt and bile stress. J. Appl. Microbiol..

[90] Finn S., Condell O., McClure P., Amézquita A., Fanning S. (2013). Mechanisms of survival, responses and sources of *Salmonella* in low-moisture environments. Front. Microbiol..

[91] Hennekinne J.A., De Buyser M.L., Dragacci S. (2012). *Staphylococcus aureus* and Its Food Poisoning Toxins: Characterization and Outbreak Investigation. FEMS Microbiol. Rev..

[92] Valdramidis V., Patterson M., Linton M. (2015). Modelling the recovery of *Listeria monocytogenes* in high pressure processed simulated cured meat. Food Control.

[93] Kalburge S.S., Whitaker W.B., Boyd E.F. (2014). High-Salt Preadaptation of *Vibrio parahaemolyticus* Enhances Survival in Response to Lethal Environmental Stresses. J. Food Prot..

[94] Ma J., Xu C., Liu F., Hou J., Shao H., Yu W. (2021). Stress adaptation and cross-protection of *Lactobacillus plantarum* KLDS 1.0628. CyTA J. Food.

[95] Keto-Timonen R., Hietala N., Palonen E., Hakakorpi A., Lindström M., Korkeala H. (2016). Cold Shock Proteins: A Minireview with Special Emphasis on Csp-Family of Enteropathogenic *Yersinia*. Front. Microbiol..

[96] Schmid B., Klumpp J., Raimann E., Loessner M.J., Stephan R., Tasara T. (2009). Role of Cold Shock Proteins in Growth of *Listeria monocytogenes* under Cold and Osmotic Stress Conditions. Appl. Environ. Microbiol..

[97] Bergholz T.M., Bowen B., Wiedmann M., Boor K.J. (2012). *Listeria monocytogenes* Shows Temperature-Dependent and -Independent Responses to Salt Stress, Including Responses That Induce Cross-Protection against Other Stresses. Appl. Environ. Microbiol..

[98] De Filippis F., La Storia A., Villani F., Ercolini D. (2013). Exploring the Sources of Bacterial Spoilers in Beefsteaks by Culture-Independent High-Throughput Sequencing. PLoS ONE.

[99] Marmion M., Ferone M., Whyte P., Scannell A. (2021). The changing microbiome of poultry meat; From farm to fridge. Food Microbiol..

[100] Pellissery A.J., Vinayamohan P.G., Amalaradjou M.A.R., Venkitanarayanan K. (2019). Spoilage Bacteria and Meat Quality. Meat Quality Analysis.

[101] Kumar P., Chatli M.K., Verma A.K., Mehta N., Malav O.P., Kumar D., Sharma N. (2017). Quality, functionality, and shelf life of fermented meat and meat products: A review. Crit. Rev. Food Sci. Nutr..

[102] Quijada N.M., De Filippis F., Sanz J.J., García-Fernández M.D.C., Rodríguez-Lázaro D., Ercolini D., Hernández M. (2018). Different *Lactobacillus* populations dominate in “Chorizo de León” manufacturing performed in different production plants. Food Microbiol..

[103] Juárez-Castelán C., García-Cano I., Escobar-Zepeda A., Azaola-Espinosa A., Alvarez-Cisneros Y.M., Ponce-Alquicira E. (2019). Evaluation of the bacterial diversity of Spanish-type chorizo during the ripening process using high-throughput sequencing and physicochemical characterization. Meat Sci..

[104] Fougy L., Desmonts M.-H., Coeuret G., Fassel C., Hamon E., Hézard B., Champomier-Vergès M.-C., Chaillou S. (2016). Reducing Salt in Raw Pork Sausages Increases Spoilage and Correlates with Reduced Bacterial Diversity. Appl. Environ. Microbiol..

[105] Ortega G., Laín A., Tadeo X., López-Méndez B., Castaño D., Millet O. (2011). Halophilic enzyme activation induced by salts. Sci. Rep..

[106] Sekar A., Kim K., Kim S. (2020). Halophilic Bacteria in the Food Industry. Encyclopedia of Marine Biotechnology.

[107] Casaburi A., Piombino P., Nychas G.-J., Villani F., Ercolini D. (2015). Bacterial populations and the volatilome associated to meat spoilage. Food Microbiol..

[108] Doulgeraki A.I., Ercolini D., Villani F., Nychas G.-J.E. (2012). Spoilage microbiota associated to the storage of raw meat in different conditions. Int. J. Food Microbiol..

[109] Stellato G., La Storia A., De Filippis F., Borriello G., Villani F., Ercolini D. (2016). Overlap of Spoilage-Associated Microbiota between Meat and the Meat Processing Environment in Small-Scale and Large-Scale Retail Distributions. Appl. Environ. Microbiol..

[110] Hwang B.K., Choi H., Choi S.H., Kim B.-S. (2020). Analysis of Microbiota Structure and Potential Functions Influencing Spoilage of Fresh Beef Meat. Front. Microbiol..

[111] Fadda S., López C., Vignolo G. (2010). Role of lactic acid bacteria during meat conditioning and fermentation: Peptides generated as sensorial and hygienic biomarkers. Meat Sci..

[112] Cui S., Fan Z., Chen W. (2019). Lactic Acid Bacteria and Fermented Meat Products. Lactic Acid Bacteria.

[113] Castilho N.P.A., Colombo M., De Oliveira L.L., Todorov S.D., Nero L.A. (2019). *Lactobacillus* curvatus UFV-NPAC1 and other lactic acid bacteria isolated from calabresa, a fermented meat product, present high bacteriocinogenic activity against *Listeria monocytogenes*. BMC Microbiol..

[114] Komora N., Maciel C., Amaral R.A., Fernandes R., Castro S.M., Saraiva J.A., Teixeira P. (2021). Innovative hurdle system towards *Listeria monocytogenes* inactivation in a fermented meat sausage model—High pressure processing assisted by bacteriophage P100 and bacteriocinogenic Pediococcus acidilactici. Food Res. Int..

[115] Laranjo M., Gomes A., Agulheiro-Santos A.C., Potes M.E., Cabrita M.J., Garcia R., Rocha J.M., Roseiro L.C., Fernandes M.J., Fraqueza M.J. (2017). Impact of salt reduction on biogenic amines, fatty acids, microbiota, texture and sensory profile in traditional blood dry-cured sausages. Food Chem..

[116] Sleator R.D., Hill C. (2007). Food reformulations for improved health: A potential risk for microbial food safety?. Med. Hypotheses.

[117] Carrasco E., Valero A., Pérez-Rodríguez F., García-Gimeno R., Zurera G. (2007). Management of microbiological safety of ready-to-eat meat products by mathematical modelling: *Listeria monocytogenes* as an example. Int. J. Food Microbiol..

[118] Uyttendaele M., Busschaert P., Valero A., Geeraerd A., Vermeulen A., Jacxsens L., Goh K., De Loy A., Van Impe J., Devlieghere F. (2009). Prevalence and challenge tests of *Listeria monocytogenes* in Belgian produced and retailed mayonnaise-based deli-salads, cooked meat products and smoked fish between 2005 and 2007. Int. J. Food Microbiol..

[119] Iannetti L., Salini R., Sperandii A.F., Santarelli G.A., Neri D., Di Marzio V., Romantini R., Migliorati G., Baranyi J. (2017). Predicting the kinetics of *Listeria monocytogenes* and *Yersinia enterocolitica* under dynamic growth/death-inducing conditions, in Italian style fresh sausage. Int. J. Food Microbiol..

[120] Leães Y.S.V., Silva J.S., Robalo S.S., Pinton M.B., dos Santos S.P., Wagner R., Brasil C.C.B., de Menezes C.R., Barin J.S., Campagnol P.C.B. (2021). Combined effect of ultrasound and basic electrolyzed water on the microbiological and oxidative profile of low-sodium mortadellas. Int. J. Food Microbiol..

[121] Perea-Sanz L., Montero R., Belloch C., Flores M. (2018). Nitrate reduction in the fermentation process of salt reduced dry sausages: Impact on microbial and physicochemical parameters and aroma profile. Int. J. Food Microbiol..

[122] Pietrasik Z., Gaudette N.J. (2015). The effect of salt replacers and flavor enhancer on the processing characteristics and consumer acceptance of turkey sausages. J. Sci. Food Agric..

[123] Peruzy M.F., Houf K., Joossens M., Yu Z., Proroga Y.T.R., Murru N. (2021). Evaluation of microbial contamination of different pork carcass areas through culture-dependent and independent methods in small-scale slaughterhouses. Int. J. Food Microbiol..

[124] Fraqueza M.J., Barreto A.S. (2015). HACCP: Hazard Analysis and Critical Control Points. Handbook of Fermented Meat and Poultry.

[125] Omer M.K., Alvarez-Ordóñez A., Prieto M., Skjerve E., Asehun T., Alvseike O.A. (2018). A Systematic Review of Bacterial Foodborne Outbreaks Related to Red Meat and Meat Products. Foodborne Pathog. Dis..

[126] Hazards E.P.O.B. (2015). Scientific Opinion on the development of a risk ranking toolbox for the EFSA BIOHAZ Panel. EFSA J..

[127] EFSA (2021). The European Union One Health 2020 Zoonoses Report. EFSA J..

[128] Arguello H., Álvarez-Ordoñez A., Carvajal A., Rubio P., Prieto M. (2013). Role of Slaughtering in Salmonella Spreading and Control in Pork Production. J. Food Prot..

[129] Akil L., Ahmad H.A. (2019). Quantitative Risk Assessment Model of Human Salmonellosis Resulting from Consumption of Broiler Chicken. Diseases.

[130] Thames H.T., Sukumaran A.T. (2020). A Review of *Salmonella* and *Campylobacter* in Broiler Meat: Emerging Challenges and Food Safety Measures. Foods.

[131] Bonardi S., Bruini I., Bolzoni L., Cozzolino P., Pierantoni M., Brindani F., Bellotti P., Renzi M., Pongolini S. (2017). Assessment of *Salmonella* survival in dry-cured Italian salami. Int. J. Food Microbiol..

[132] Morales-Partera Á.M., Cardoso-Toset F., Jurado-Martos F., Astorga R.J., Huerta B., Luque I., Tarradas C., Gómez-Laguna J. (2017). Survival of selected foodborne pathogens on dry cured pork loins. Int. J. Food Microbiol..

[133] Shiroda M., Pratt Z.L., Döpfer D., Wong A.C., Kaspar C.W. (2014). RpoS impacts the lag phase of *Salmonella enterica* during osmotic stress. FEMS Microbiol. Lett..

[134] Heir E., Holck A., Omer M., Alvseike O., Høy M., Mage I., Axelsson L. (2010). Reduction of verotoxigenic *Escherichia coli* by process and recipe optimisation in dry-fermented sausages. Int. J. Food Microbiol..

[135] Sartz L., De Jong B., Hjertqvist M., Plym-Forshell L., Alsterlund R., Löfdahl S., Osterman B., Ståhl A., Eriksson E., Hansson H.B. (2008). An Outbreak of *Escherichia coli* O157:H7 Infection in Southern Sweden Associated with Consumption of Fermented Sausage; Aspects of Sausage Production That Increase the Risk of Contamination. Epidemiol. Infect..

[136] European Food Safety Authority (EFSA), European Centre for Disease Prevention and Control (ECDC) (2018). The European Union summary report on trends and sources of zoonoses, zoonotic agents and food-borne outbreaks in 2017. EFSA J..

[137] Raimann E., Schmid B., Stephan R., Tasara T. (2009). The Alternative Sigma Factor Sigma(L) of *L. monocytogenes* Promotes Growth under Diverse Environmental Stresses. Foodborne Pathog. Dis..

[138] Medveov A., Valk L., Eissa A. (2012). Ubomr *Staphylococcus aureus*: Characterisation and Quantitative Growth Description in Milk and Artisanal Raw Milk Cheese Production. Structure and Function of Food Engineering.

[139] Sihto H.-M., Stephan R., Engl C., Chen J., Johler S. (2017). Effect of food-related stress conditions and loss of agr and sigB on seb promoter activity in *S. aureus*. Food Microbiol..

[140] Peck M.W., van Vliet A.H. (2016). Impact of *Clostridium botulinum* genomic diversity on food safety. Curr. Opin. Food Sci..

[141] Courtney-Long E., Carrol D., Zhang Q., Stevens A., Griffin-Blake S., Armour B., Campbell V. (2015). Prevalence of Disability and Disability Type Among Adults—United States 2013. MMWR Morb. Mortal. Wkly. Rep..

[142] Laranjo M., Gomes A., Agulheiro-Santos A.C., Potes M.E., Cabrita M.J., Garcia R., Rocha J.M., Roseiro L.C., Fernandes M.J., Fraqueza M.H. (2016). Characterisation of “Catalão” and “Salsichão” Portuguese traditional sausages with salt reduction. Meat Sci..

[143] Cittadini A., Domínguez R., Gómez B., Pateiro M., Pérez-Santaescolástica C., López-Fernández O., Sarriés M.V., Lorenzo J.M. (2020). Effect of NaCl replacement by other chloride salts on physicochemical parameters, proteolysis and lipolysis of dry-cured foal “cecina”. J. Food Sci. Technol..

[144] Khan I., Tango C.N., Miskeen S., Lee B.H., Oh D.H. (2017). Hurdle Technology: A Novel Approach for Enhanced Food Quality and Safety—A Review. Food Control.

[145] Shan L.C., Regan A., Monahan F.J., Li C., Murrin C., Lalor F., Wall P.G., McConnon A. (2016). Consumer views on “healthier” processed meat. Br. Food J..

[146] Devlieghere F., Vermeiren L., Debevere J. (2004). New preservation technologies: Possibilities and limitations. Int. Dairy J..

[147] Sadaghiani S.K., Aliakbarlu J., Tajik H., Mahmoudian A. (2019). Anti-listeria activity and shelf life extension effects of *Lactobacillus* along with garlic extract in ground beef. J. Food Saf..

[148] Castellano P., Peña N., Ibarreche M.P., Carduza F., Soteras T., Vignolo G. (2018). Antilisterial efficacy of *Lactobacillus* bacteriocins and organic acids on frankfurters. Impact on sensory characteristics. J. Food Sci. Technol..

[149] Hu Z.Y., Balay D., Hu Y., McMullen L.M., Gänzle M.G. (2019). Effect of chitosan, and bacteriocin—Producing *Carnobacterium maltaromaticum* on survival of *Escherichia coli* and *Salmonella Typhimurium* on beef. Int. J. Food Microbiol..

[150] Kumar Y., Kaur K., Shahi A.K., Kairam N., Tyagi S.K. (2017). Antilisterial, antimicrobial and antioxidant effects of pediocin and *Murraya koenigii* berry extract in refrigerated goat meat emulsion. LWT Food Sci. Technol..

[151] Yang H.-J., Wang H.-F., Tao F., Li W.-X., Cao G.-T., Yang Y.-Y., Xu X.-L., Zhou G.-H., Shen Q. (2021). Structural basis for high-pressure improvement in depolymerization of interfacial protein from RFRS meat batters in relation to their solubility. Food Res. Int..

[152] Xiao Q., Xu M., Xu B., Chen C., Deng J., Li P. (2021). Combined Effect of High-Pressure Processing with Spice Extracts on Quality of Low-Salt Sausage during Refrigerated Storage. Foods.

[153] Horita C.N., Baptista R.C., Caturla M.Y., Lorenzo J.M., Barba F.J., Sant’Ana A.S. (2018). Combining reformulation, active packaging and non-thermal post-packaging decontamination technologies to increase the microbiological quality and safety of cooked ready-to-eat meat products. Trends Food Sci. Technol..

[154] Grossi A., Søltoft-Jensen J., Knudsen J.C., Christensen M., Orlien V. (2012). Reduction of salt in pork sausages by the addition of carrot fibre or potato starch and high pressure treatment. Meat Sci..

[155] Hygreeva D., Pandey M. (2016). Novel approaches in improving the quality and safety aspects of processed meat products through high pressure processing technology—A review. Trends Food Sci. Technol..

[156] Duranton F., Guillou S., Simonin H., Chéret R., de Lamballerie M. (2012). Combined use of high pressure and salt or sodium nitrite to control the growth of endogenous microflora in raw pork meat. Innov. Food Sci. Emerg. Technol..

[157] Stollewerk K., Jofré A., Comaposada J., Arnau J., Garriga M. (2012). The effect of NaCl-free processing and high pressure on the fate of *Listeria monocytogenes* and *Salmonella* on sliced smoked dry-cured ham. Meat Sci..

[158] Hammou F.B., Skali S.N., Idaomar M., Abrini J. (2010). Combinations of Nisin with Salt (NaCl) to Control *Listeria monocytogenes* on Sheep Natural Sausage Casings Stored at 6 °C. African J. Biotechnol..

[159] Ojha K.S., Keenan D.F., Bright A., Kerry J.P., Tiwari B. (2016). Ultrasound assisted diffusion of sodium salt replacer and effect on physicochemical properties of pork meat. Int. J. Food Sci. Technol..

[160] Barros J.C., Gois T.S., Pires M.A., Rodrigues I., Trindade M.A. (2019). Sodium reduction in enrobed restructured chicken nuggets through replacement of NaCl with CaCl_2_. J. Food Sci. Technol..

[161] Pandya J.K., Decker K.E., Goulette T., Kinchla A.J. (2020). Sodium reduction in Turkey breast meat by using sodium anion species. LWT Food Sci. Technol..

[162] Aliño M., Grau R., Toldrá F., Blesa E., Pagán M.J., Barat J.M. (2010). Physicochemical properties and microbiology of dry-cured loins obtained by partial sodium replacement with potassium, calcium and magnesium. Meat Sci..

[163] Blesa E., Aliño M., Barat J.M., Grau R., Toldrá F., Pagán M. (2008). Microbiology and physico-chemical changes of dry-cured ham during the post-salting stage as affected by partial replacement of NaCl by other salts. Meat Sci..

[164] Harper N.M., Getty K.J. (2012). Effect of Salt Reduction on Growth of *Listeria monocytogenes* in Meat and Poultry Systems. J. Food Sci..

[165] Shadbolt C., Ross T., McMeekin T. (2001). Differentiation of the effects of lethal pH and water activity: Food safety implications. Lett. Appl. Microbiol..

[166] Devlieghere F., Vermeiren L., Bontenbal E., Lamers P.-P., Debevere J. (2009). Reducing salt intake from meat products by combined use of lactate and diacetate salts without affecting microbial stability. Int. J. Food Sci. Technol..

[167] Lorenzo J.M., Cittadini A., Bermúdez R., Munekata P.E., Domínguez R. (2015). Influence of partial replacement of NaCl with KCl, CaCl_2_ and MgCl_2_ on proteolysis, lipolysis and sensory properties during the manufacture of dry-cured lacón. Food Control.

[168] Samapundo S., Ampofo-Asiama J., Anthierens T., Xhaferi R., Van Bree I., Szczepaniak S., Goemaere O., Steen L., Dhooge M., Paelinck H. (2010). Influence of NaCl reduction and replacement on the growth of *Lactobacillus* sakei in broth, cooked ham and white sauce. Int. J. Food Microbiol..

[169] Rios-Mera J.D., Saldaña E., Patinho I., Selani M.M., Contreras-Castillo C.J. (2021). Enrichment of NaCl-reduced burger with long-chain polyunsaturated fatty acids: Effects on physicochemical, technological, nutritional, and sensory characteristics. Meat Sci..

[170] Vidal V.A., Biachi J.P., Paglarini C., Pinton M.B., Campagnol P.C., Esmerino E.A., da Cruz A.G., Morgano M.A., Pollonio M.A. (2019). Reducing 50% sodium chloride in healthier jerked beef: An efficient design to ensure suitable stability, technological and sensory properties. Meat Sci..

[171] França F., Harada-Padermo S.D.S., Frasceto R.A., Saldaña E., Lorenzo J.M., Vieira T.M.F.D.S., Selani M.M. (2022). Umami ingredient from shiitake (*Lentinula edodes*) by-products as a flavor enhancer in low-salt beef burgers: Effects on physicochemical and technological properties. LWT.

[172] Gaudette N.J., Pietrasik Z. (2017). The sensory impact of salt replacers and flavor enhancer in reduced sodium processed meats is matrix dependent. J. Sens. Stud..

[173] Horita C., Morgano M.A., Celeghini R., Pollonio M. (2011). Physico-chemical and sensory properties of reduced-fat mortadella prepared with blends of calcium, magnesium and potassium chloride as partial substitutes for sodium chloride. Meat Sci..

[174] García-Lomillo J., González-SanJosé M.A.L., Del Pino-García R., Rivero-Pérez M.A.D., Muñiz-Rodríguez P. (2017). Alternative natural seasoning to improve the microbial stability of low-salt beef patties. Food Chem..

[175] Omer M., Prieto B., Rendueles E., Alvarez-Ordóñez A., Lunde K., Alvseike O., Prieto M. (2015). Microbiological, physicochemical and sensory parameters of dry fermented sausages manufactured with high hydrostatic pressure processed raw meat. Meat Sci..

[176] Rendueles E., Omer M., Alvseike O., Alonso-Calleja C., Capita R., Prieto M. (2011). Microbiological food safety assessment of high hydrostatic pressure processing: A review. LWT.

[177] Aymerich T., Picouet P., Monfort J. (2008). Decontamination technologies for meat products. Meat Sci..

[178] Bajovic B., Bolumar T., Heinz V. (2012). Quality considerations with high pressure processing of fresh and value added meat products. Meat Sci..

[179] Hereu A., Dalgaard P., Garriga M., Aymerich T., Bover-Cid S. (2014). Analysing and modelling the growth behaviour of *Listeria monocytogenes* on RTE cooked meat products after a high pressure treatment at 400 MPa. Int. J. Food Microbiol..

[180] Bover-Cid S., Belletti N., Garriga M., Aymerich T. (2011). Model for *Listeria monocytogenes* inactivation on dry-cured ham by high hydrostatic pressure processing. Food Microbiol..

[181] Rubio B., Possas A., Rincón F., García-Gímeno R.M., Martínez B. (2018). Model for *Listeria monocytogenes* inactivation by high hydrostatic pressure processing in Spanish chorizo sausage. Food Microbiol..

[182] Luckose F., Pandey M.C., Chauhan O.P., Sultana K., Abhishek V. (2015). Effect of High Pressure Processing on the Quality Characteristics and Shelf Life of Low-Sodium Re-Structured Chicken Nuggets. J. Food Nutr. Res..

[183] EURL Lm (2021). EURL Lm Technical Guidance Document on Challenge Tests and Durability Studies for Assessing Shelf-Life of Ready-to-Eat Foods Related to Listeria monocytogenes.

[184] Nikodinoska I., Baffoni L., Di Gioia D., Manso B., García-Sánchez L., Melero B., Rovira J. (2019). Protective cultures against foodborne pathogens in a nitrite reduced fermented meat product. LWT.

[185] Bernardo R., Barreto A.S., Nunes T., Henriques A.R. (2020). Estimating *Listeria monocytogenes* Growth in Ready-to-Eat Chicken Salad Using a Challenge Test for Quantitative Microbial Risk Assessment. Risk Anal..

[186] Di Gioia D., Mazzola G., Nikodinoska I., Aloisio I., Langerholc T., Rossi M., Raimondi S., Melero B., Rovira J. (2016). Lactic acid bacteria as protective cultures in fermented pork meat to prevent *Clostridium* spp. growth. Int. J. Food Microbiol..

[187] Bonilauri P., Merialdi G., Ramini M., Bardasi L., Taddei R., Grisenti M.S., Daminelli P., Cosciani-Cunico E., Dalzini E., Frustoli M.A. (2021). Modeling the behavior of *Listeria innocua* in Italian salami during the production and high-pressure validation of processes for exportation to the U.S. Meat Sci..

[188] Borges A.F., Cózar A., Patarata L., Gama L.T., Alfaia C.M., Fernandes M.J., Pérez H.V., Fraqueza M.J. (2020). Effect of high hydrostatic pressure challenge on biogenic amines, microbiota, and sensory profile in traditional poultry- and pork-based semidried fermented sausage. J. Food Sci..

[189] Álvarez-Ordóñez A., Leong D., Hickey B., Beaufort A., Jordan K. (2015). The challenge of challenge testing to monitor *Listeria monocytogenes* growth on ready-to-eat foods in Europe by following the European Commission (2014) Technical Guidance document. Food Res. Int..

[190] Palanichamy A., Jayas D.S., Holley R.A. (2008). Predicting Survival of *Escherichia coli* O157:H7 in Dry Fermented Sausage Using Artificial Neural Networks. J. Food Prot..

[191] Skjerdal T., Gangsei L.E., Alvseike O., Kausrud K., De Cesare A., Alexa E.-A., Alvarez-Ordóñez A., Moen L.H., Osland A.M., From C. (2021). Development and validation of a regression model for *Listeria monocytogenes* growth in roast beefs. Food Microbiol..

[192] Hereu A., Dalgaard P., Garriga M., Aymerich T., Bover-Cid S. (2012). Modeling the high pressure inactivation kinetics of *Listeria monocytogenes* on RTE cooked meat products. Innov. Food Sci. Emerg. Technol..

[193] Huang L. (2016). Evaluating the Performance of a New Model for Predicting the Growth of *Clostridium perfringens* in Cooked, Uncured Meat and Poultry Products under Isothermal, Heating, and Dynamically Cooling Conditions. J. Food Sci..

[194] Bover-Cid S., Belletti N., Aymerich T., Garriga M. (2015). Modeling the protective effect of a w and fat content on the high pressure resistance of *Listeria monocytogenes* in dry-cured ham. Food Res. Int..

[195] Gunvig A., Borggaard C., Hansen F., Hansen T.B., Aabo S. (2016). ConFerm—A tool to predict the reduction of pathogens during the production of fermented and matured sausages. Food Control.

[196] Hansen T.B., Abdalas S., Al-Hilali I., Hansen L.T. (2021). Predicting the effect of salt on heat tolerance of *Listeria monocytogenes* in meat and fish products. Int. J. Food Microbiol..

[197] Alía A., Rodríguez A., Andrade M.J., Gómez F.M., Córdoba J.J. (2019). Combined effect of temperature, water activity and salt content on the growth and gene expression of *Listeria monocytogenes* in a dry-cured ham model system. Meat Sci..

[198] Qu K., Guo F., Liu X., Lin Y., Zou Q. (2019). Application of Machine Learning in Microbiology. Front. Microbiol..

